# Views of Community Pharmacists on Antimicrobial Resistance and Antimicrobial Stewardship in Jordan: A Qualitative Study

**DOI:** 10.3390/antibiotics10040384

**Published:** 2021-04-03

**Authors:** Doaa Saleh, Rana Abu-Farha, Tareq L. Mukattash, Muna Barakat, Eman Alefishat

**Affiliations:** 1 Department of Clinical Pharmacy and Therapeutics, Faculty of Pharmacy, Applied Science Private University, Amman 11937, Jordan; Dodo_s992@outlook.com (D.S.); r_abufarha@asu.edu.jo (R.A.-F.); m_barakat@asu.edu.jo (M.B.); 2 Department of Clinical Pharmacy, Faculty of Pharmacy, Jordan University of Science and Technology, Irbid 22110, Jordan; tlmukattash@just.edu.jo; 3 Department Biopharmaceutics and Clinical Pharmacy, Faculty of Pharmacy, The University of Jordan, Amman 11937, Jordan; 4 Department of Pharmacology, College of Medicine and Health Science, Khalifa University of Science and Technology, Abu Dhabi 127788, United Arab Emirates; 5 Center for Biotechnology, Khalifa University of Science and Technology, Abu Dhabi 127788, United Arab Emirates

**Keywords:** antimicrobial stewardship, antimicrobial resistance, community pharmacist, qualitative research, Jordan

## Abstract

The Center for Disease Control and Prevention and the World Health Organization issued a practical approach and Global Action Plan to control the threatening emerging antibacterial resistance. One of the main bases of this plan is the Antimicrobial Stewardship Program (ASPs). This study aimed to evaluate community pharmacists’ awareness and perception towards antimicrobial resistance and ASPs in Jordan. Thus, a qualitative study was conducted through in-depth interviews with twenty community pharmacists. Convenience sampling was used in the study. Qualitative analysis of the data yielded four themes and eleven sub-themes. All the respondents showed a good understanding of the causes of antimicrobial resistance. The most important cause reported by them was the non-restricted prescription of antimicrobials. Most of the pharmacists believed that they are competent to provide ASPs, however, they believed that there are several barriers against the implementation of ASPs in community pharmacies in Jordan. Barriers demonstrated by the pharmacists, included organizational obstacles, resources obstacles, and personal obstacles. In conclusion, this study revealed several barriers against the implementation of ASPs in community pharmacies in Jordan. Incorporating ASPs in the community pharmacy settings requires proper pharmacist training, several academic disciplines team efforts, and good pharmacy practice of antimicrobial guidelines.

## 1. Introduction

Nowadays, the whole world is facing a serious disaster of multi-drug resistance (MDR) microorganisms, which is associated with increased rates of morbidity and mortality [1]. The global reports estimated the rate of MDR-caused death in the United States and European Union by around 2.6 million/year, while in the developing countries (e.g., Southern Asia) the case is worse and the annual MDR-death rate exceeded the United State/Europe by more than three folds [2,3]. Such alarming numbers shed the light on many possible reasons including the low-middle socioeconomic status of the population and limited accessibility to healthcare services to conduct the proper microbiological investigational tests then to get suitable treatment [4].

Therefore, the prevalence of the MDR phenomenon urges the need for appropriate solutions such as a novel antimicrobial drug discovery with new mechanisms of action [5]. However, this strategy was not enough to solve the problem [6], which pushed the WHO to release a Global Action Plan (GAP) in 2015 to control the MDR threat [7]. One of the essential pillars of GAP is the Antimicrobial Stewardship Programs (ASPs), which involve the correct use of antimicrobials among humans and animals [7,8]. The main aim of ASPs is to manage and optimize the microbial infections’ clinical outcomes with limited consequences of antimicrobial therapy [9,10]. Those consequences could be related to drug toxicity, side effects, cost-effectiveness [9,10].

In developing countries, several studies have demonstrated improper practice regarding antimicrobial handling [11,12,13]. As, a significant number of antimicrobials are used to be dispensed without prescription in the community pharmacy settings due to poor enforcement of regulations, poverty, unaffordable cost of medical consultation, and lack of health insurance [11,12,13,14]. Unfortunately, such practice is implicated in the emergence of bacterial resistance and complicates the treatment strategies [15,16]. Considering the necessity for the management of antimicrobials misuse, many countries had adopted the ASP guidelines [17,18]. However, the success in the implementation of ASPs was demonstrated obviously in hospitals rather than community pharmacies (which is considered a major source of antimicrobials misuse) [19,20].

In Jordan’s context, it is considered a low middle-income country with a population of almost 10,260,000 in 2020 [21]. In Jordan, public and private sectors are the two main sectors in the healthcare system including hospitals, primary care clinics, pharmacies [12,13]. The Jordan Pharmacists Association (JPA) is the main Jordan pharmacy professional organization, established in 1957, with about 25,700 member pharmacists (October 2020) [14,22]. According to the latest statistics (2020) by JPA, there are 3500 community pharmacies across the country [22]. For all pharmacists, their membership in JPA is mandatory, which means that pharmacists cannot practice in Jordan unless they are registered with the JPA [23]. The role of community pharmacists in Jordan executes various responsibilities include delivering a wide range of non-prescription and prescription medicines [24]. Besides, the responsibilities of most of them remain focused on dispensing medications, inventory control, and execute physician orders [24]. Patient counseling and education are rarely performed effectively, and very few pharmacies keep any patient records [24]. Legislation in Jordan requires that prescription drugs are only dispensed by licensed pharmacists [25]. Additionally, the Jordan Food and Drug Administration (JFDA) regulations confirm that antibiotics should not be dispensed without a prescription. Therefore, despite the availability of antibiotics use policy, a lack of enforcement of these policies was reported [26]. Regulatory authorities are advised to reverse the trend of antibiotics resistance by enforcing the regulations related to antibiotics which will improve current practices related to antibiotic use [20].

Noteworthy, the Jordanian Ministry of Health issued the national antimicrobial resistance surveillance system which is supported by WHO and followed the Global Antimicrobial Resistance Surveillance System (GLASS), as a preliminary step for strengthening global and national surveillance systems [27]. They are working hand in hand with JFDA and JPA to support the implementation of these regulations [26]. Despite all of those efforts, there is a clear problem in the commitment of health care providers (mainly community pharmacists) to these regulations and protocols. Studies reported that community pharmacists are usually unaware of ASPs and the number of confronted obstacles overcomes the ability of ASPs application [20]. Apparently, there are no sufficient studies that have evaluated the pharmacists’ awareness and perceptions towards ASPs in Jordan. Therefore, this study aimed to evaluate the pharmacists’ awareness and perception towards antimicrobial resistance and ASPs in Jordan. As the findings of this study could come up with valuable recommendations for healthcare policymakers and enhance the fruitful implementation of ASPs at Jordan.

## 2. Methods

### 2.1. Study Design, Setting, and Instrument

A qualitative design was used for this study to explore community pharmacists’ awareness and perception regarding antimicrobials resistance and antimicrobial stewardship program in Jordan. Any community pharmacists registered in the pharmacist’s syndicate, having at least one year of experience in retail drug outlets, and willing to participate in this study were eligible for inclusion. In this study, a pragmatic sample of eligible community pharmacists was invited to take part in this study using WhatsApp invitation. Pharmacists listed in a WhatsApp group of community pharmacists who accredited to provide community training for pharmacy students at Applied Science Private University were invited to participate in this study, and they were informed that interviews will be conducted using Zoom Application for safety consideration due to COVID-19 pandemic.

### 2.2. Development of the Interview Guide

A semi-structured interview guide (Supplementary Material) was established and developed by four investigators from the research team who have previous experience in performing qualitative research. Questions of the interview were designed to be open-ended discussion questions. The interview guide was evaluated by conducting a pilot-tested interview with two community pharmacists to ensure questions’ reliability and understandability. The questions were improved according to their feedback. The interviews of the pilot study were not included in the final analysis of this study.

### 2.3. Data Collection

Interviews were conducted one-to-one using the Zoom application between October and November 2020. Interviews were scheduled with participants after obtaining their electronic informed consent sent to them via WhatsApp. Each interview was recorded after taking participants’ permission. All interviews with pharmacists were carried out in Arabic. Each interview started with questions to elicit socio-demographic information followed by a set of questions that explored: (1) causes of antimicrobial resistance, (2) perception toward the role of pharmacists in ASPs, (3) barriers against the implementation of ASPs, and (4) suggestions to enhance the implementation of ASPs. The interview was based on the interview guide, and participants were given the liberty to discuss any additional points regarding the research.

### 2.4. Ethical Considerations

Ethical approval was obtained from the Institutional Review Board at Applied Science Private University (approval number 2020-PHA-20). Participants were requested to approve an electronic informed consent. Moreover, they were informed that their participation in the study is voluntary, and they were assured that their responses would be kept confidential and anonymous.

### 2.5. Data Analysis

All interviews in this study were audio-taped, and (D.S.) transcribed each of these interviews. To guarantee content consistency, two independent expert translators translated transcripts into English and then back-translated to Arabic; we then used the English version through the analysis. The researchers thoroughly and independently read all transcripts; topics raised in each interview were then summarized into notes. Utilizing a thematic content analysis approach described by Patton [28], all authors then reviewed notes from each interview and turned them into all potential themes. All resultant themes were then evaluated, and, whenever appropriate, themes were renamed or grouped together due to similarity.

As we continued the assessment, following the approach described by Pope et al. [29], content analysis was performed where specific attention was paid to any response that concluded any existing themes. We then pursued alternative explanations aiming to confirm the concluded themes we identified from each interview. In addition, we examined the commonality of themes across all the interviews to check for any patterns in participants’ responses. In the case of new themes emerging, the similarity was rechecked, and some themes were either categorized or gathered together, whatever was agreed to be more appropriate. Finally, (R. A.) independently analyzed the transcriptions, and after all, researchers reached an agreement, the concluded themes were then confirmed as the participants’ responses.

For socio-demographic information, data was analyzed using thestatistical package for social science (SPSS) version 22 (SPSS Inc., Chicago, IL, USA). The descriptive analysis was done using mean/standard deviation (SD) or median/Interquartile range (IQR) for continuous variables and frequency/percentage for qualitative variables.

## 3. Results

### 3.1. Sociodemographic Characteristics of the Study Sample

A total of 20 community pharmacists were interviewed, and the duration of the interview ranged from 10–47 min, with a mean of 27.1 (SD = 10.6) minutes. Participants’ have a median age of 25.8 (IQR = 8.0) years and a community practice experience of 4.5 (IRQ = 6.0) years.

Most of the participants were female (n = 14, 70%), and 60% of them (n = 12) were employees. The majority of them (n = 16, 80%) worked in independent community pharmacies. Three-third of the participants (n = 15, 75%) revealed that they serve more than 50 patients/day, while 30% of them (n = 6) reported attending previous workshops about ASPs. The demographic characteristics of the participants are outlined in Table 1.

### 3.2. Thematic Analysis

#### 3.2.1. Causes of Antimicrobial Resistance

Qualitative data analysis was performed to cover four main domains: causes of antimicrobial resistance, perception towards the role of pharmacists in ASPs, barriers against the implementation of ASPs, and suggestions to enhance the implementation of ASPs. The emerged themes and sub-themes are summarized in Table 2.

Qualitative analysis for antimicrobial resistance causes revealed two main emerging themes: **lack of appropriate knowledge about antimicrobial agents and non-restricted prescription of antimicrobials**.

The first theme was the **lack of appropriate knowledge about antimicrobial agents**. Respondents raised serious concerns and doubt about pharmacists’ ability to dispense antimicrobials correctly because of the lack of knowledge about antimicrobials, especially by old graduates. They believed that this is among the main causes of antimicrobial resistance *“There are many factors that may cause antimicrobial resistance; weakness in knowledge is a possible reason, and many pharmacists graduated a long time ago and their information is not updated.”* Moreover, some pharmacists believed that the boring pharmacy profession does not encourage them to up-date their knowledge *“Pharmacist in Jordan has gone through cycles of boring and few pharmacists seeking to develop their knowledge”.*

The second theme was the **non-restricted prescription of antimicrobials**. Sub-themes were **lack of law enforcement, physicians’ malpractice, pharmacists’ malpractice, and incorrect patients’ beliefs and practice**.

The first sub-theme was the **lack of law enforcement**. The majority of respondents believed that the main reason for antimicrobial resistance in Jordan is that antimicrobials are being purchased without a prescription. Community pharmacists reported a lack of enforcements of laws and regulations that prohibit antimicrobials dispensing without a prescription in Jordan *“The dispensing of antimicrobials without prescription was not prevented in Jordan, and it was among the things suggested, but there is no decision regarding it.”* And this may be the main reason for the emerging antimicrobial resistance problem *“I believe that there is no law in Jordan prohibits over-the-counter dispensing of antimicrobials, and there is no control of the dispensing of antimicrobials.”*

The second sub-theme was **malpractice by physicians**. Respondents pointed out that a physicians’ malpractice is a serious issue that would increase antimicrobial resistance. They believed that pressure imposed by patients is the main reason that makes physicians dispense unnecessary antimicrobials for patients *“The second reason is that some doctors always prescribe the antimicrobial whatever the symptoms are, and they say that if we don’t fill the prescription with antimicrobials, the patient will complain.”.* Moreover, pharmacists pointed out that physicians support to some pharmaceutical manufacturing represents the main reason that would increase the emergence of antimicrobial resistance *“doctors dispense antimicrobial for the support of certain pharmaceutical company than other companies and they write antimicrobials in the prescription more than the patient needs.”*

The third sub-theme is the **malpractice of pharmacists.** The majority of respondents believed that increasing pharmacies’ profit was among the main reasons for overprescribing antimicrobials in community pharmacies *“another reason is that some pharmacists want to increase the profit by dispensing antimicrobials and unfortunately they dispense broad-spectrum antimicrobial with higher price”.* Moreover, community pharmacists raised serious malpractice that some pharmacy owners in Jordan hire people who are not pharmacists to work in the evening shift, which may have a dangerous effect on antimicrobial dispensing *“another problem is the invaders of the profession who are working in a pharmacy in the evening shift, and they are not pharmacists”*.

The fourth sub-theme is the **incorrect patients’ beliefs and practices**. Respondents stated that most of the patients insisted to take antimicrobials, whatever the symptoms are, which could contribute to the emergence of antimicrobial resistance *“some patients ask for antimicrobials by name, and they diagnose themselves, and if pharmacists try to convince them not to take the antimicrobial, they insist to take the antimicrobial”.* Moreover, pharmacists believed that the lack of adherence to medication instruction by patients is another main cause *“The first thing that may cause antimicrobial resistance is the inappropriate use of antimicrobials by patients and the failure to understand that they must complete the course of medication. As soon as they get better within three days, they stop taking the drug”.*

#### 3.2.2. Perception towards the Role of Pharmacists in ASPs

The only emerging theme regarding the perception towards pharmacist’s role in ASPs was **pharmacists are competent to provide ASPs**. Respondent stated that the community pharmacists have a crucial role in ASPs because they believed that pharmacists are experts in drugs and antimicrobials dispensing *“Pharmacists are expert in drug interactions, drug information, drug effects and dosages. To some extent, antimicrobial stewardship implementation is the responsibility of pharmacists more than the responsibility of doctors.”.*

#### 3.2.3. Barriers against the Implementation of ASPs

Regarding the barriers against the implementation of ASPs, three main themes were identified, including **organizational obstacles, resources obstacles**, and **personal obstacles**.

The first theme was the **organizational obstacle**, which included two sub-themes. The first sub-theme was the **lack of law enforcement**. Community pharmacists reported that one of the barriers against applying ASPs in community pharmacies is the lack of law enforcement in Jordan that prevent pharmacists from dispensing antimicrobials without prescription *“The dispensing of antimicrobials without prescription was not prevented in Jordan, and it was among the things suggested, but there is no decision regarding it.”*. The second sub-theme was **hierarchal influence by pharmacy owners**. Most respondents stated that there is a huge pressure imposed by the owner of pharmacies on their employees to increase pharmacies’ profit, which may impede the implementation of ASPs correctly *“the owners of pharmacies are forcing their employees to use certain antimicrobials because they have a bonus on them, and subsequently, the profit of pharmacies increases.”* Also, this pressure may prevent them from developing their knowledge *“Pharmacists have many pressures in work, and there is no time left to develop their knowledge, and pharmacy owners put pressure on their employees because the most important thing for them is the profit.”.*

The second theme was **resource obstacles**, which included three sub-themes. The first sub-theme was **inadequate resources**. Some of the respondents believed that the financial capabilities in Arab countries including Jordan, may preclude the implementation of ASPs *“the application of antimicrobial stewardship requires financial capabilities, more training for pharmacists, training for doctors, and the capabilities in Arab countries do not support that”.* The second sub-theme was **lack of time**, which was reported by most of the pharmacists as a barrier against the implementation of ASPs in community pharmacies *“No, it is not applicable at the pharmacy because it will take a lot of time from pharmacists and we don’t have this time”.* The third sub-theme was **insufficiently trained pharmacists**. All community pharmacists agreed that there is a lack of proper training provided to pharmacists after graduation *“as a new graduate, we still need much training and experience to be able to dispense antimicrobials appropriately.”*

The third theme was **personal obstacles**. This theme included two subthemes. The first Sub-theme was the **patient’s socioeconomic status**. The majority of the respondents pointed out that patient financial conditions are the main factor in overprescribing antimicrobials without a prescription in community pharmacies *“some patients have difficult financial conditions, so they can’t pay for a doctor’s visit and checkup to dispense the medication. So, they will prefer to take the medication directly without prescription.”.* **The second sub-theme was profit concerns by pharmacists**. Some community pharmacists believed that the implementation of ASPs in community pharmacies would affect pharmacies’ profit *“applying antimicrobial stewardship programs would limit my sales and profit, So, I don’t want to apply them.”*

#### 3.2.4. Suggestions to Enhance the Implementation of ASPs

The only emerged theme following qualitative analysis for suggestions to enhance the implementation of ASPs was **the need for continuous professional development**. All participants stated that educational courses and workshops should be routinely applied through syndicate *“compulsory workshops should be provided for pharmacists dedicated for new information about antimicrobials”.* Moreover, they suggested the use of social media in making online courses and workshops *“Free training courses on Facebook and live broadcasting, given by experienced people, quickly and efficiently deliver the information through online educational courses”*.

## 4. Discussion

The rational use of antimicrobial agents is crucial for maintaining their efficacy against infectious diseases [30]. ASPs provide the basis for managing and optimizing the microbial infections’ clinical outcomes with limited consequences of antimicrobial therapy [30]. Community pharmacists have a prominent role in implementing ASPs and in controlling microbial resistance due to their easy accessibility by patients at retail drug outlets. The current study sheds light on community pharmacists’ understanding of antimicrobial resistance and their perceptions towards ASPs in Jordan.

Respondents to the current study reported good awareness during their interviews, suggesting several causes for antimicrobial resistance, including the lack of appropriate knowledge about antimicrobial agents and non-restricted prescription of antimicrobials. Pharmacists taking part in this study emphasized that non-prescribed antimicrobial prescription is the key factor leading to antimicrobial resistance. This mainly takes place as regulations to prescribe and dispense antimicrobials are not being reinforced in Jordan. This finding was similar to a descriptive cross-sectional study conducted on 73 community pharmacists in Lusaka, the capital city of Zambia, which stated that antimicrobial resistance is caused by the widespread non-prescription and dispensing of antimicrobials by the community pharmacists [31].

The present study’s outcomes were comparable to those of a Hungarian study that targeted community pharmacists and reported that most community pharmacists had a high level of theoretical awareness of antimicrobial resistance [32]. On the other hand, our findings were contrary to that reported by a cross-sectional survey that targeted community pharmacists, pharmacy owners, and physicians in Pakistan, which stated that community pharmacists had insufficient awareness about antimicrobial resistance [33]. Even though interviewees in this study were aware of the causes of antimicrobial resistance, this was not reflected in their practice to rationalize antimicrobial therapy. This issue was highlighted previously in a qualitative study through in-depth, semi-structured interviews for community pharmacists in Pakistan, where no relation was found between pharmacists’ awareness and their practices to reduce the use of antimicrobial agents and related resistance [20].

Respondents participating in the present study showed a positive perception about pharmacists’ role in ASPs, which aligned with earlier studies that took place in Pakistan and Ethiopia. The latter descriptive cross-sectional studies targeted community pharmacists who reported positive perceptions towards ASPs [17,34]. Respondents to our study believed that community pharmacists had a role in ASPs and have the competence to provide ASPs. Similar results were reported in a Malaysian cross-sectional survey that stated that community pharmacists were careful to take part in ASP and recognize its importance [2].

Lack of sufficient time was reported by respondents as a barrier to implementing ASPs in their workplace. Similar results were reported in a qualitative study conducted in the United States by Appaneal et al., who reported that pharmacists do not have enough time to implement ASPs [35]. Furthermore, it has been stated that the lack of proper training was an important barrier against the implementation of ASPs. This finding is in line with the outcomes of a previous study carried out to overcome the barriers to implementing ASPs, which stated that training in antimicrobial use supports the successful implementation of ASPs [36].

Dispensing antimicrobials without a prescription represents a significant issue to public health [31]. Our interviewees stated that pharmacists usually dispense antimicrobials due to the huge pressure imposed by the pharmacies owner to increase the profit of their pharmacies and that malpractice would increase antimicrobial resistance and represent a barrier against the implementation of ASPs in community pharmacies. Similar findings were reported by a previous study that took part in China, which stated that sales of antimicrobials without a prescription in low and middle-income countries account up to 93% of antimicrobial sales, and demonstrated that many factors including profit concern, pressure from the patient as well the owner of the pharmacies, and patient’s socioeconomic status who can’t afford physicians fees may contribute to dispensing antimicrobials without a prescription [37].

It was suggested that training programs and educational workshops should be provided to healthcare providers to enhance their ability to implement ASPs in community pharmacy settings. Workshops and educational courses regarding ASPs were not often offered to Jordanian community pharmacists [38]. These findings correlate with a qualitative study for community pharmacists in Bahawalpur, Pakistan, which stated that community pharmacists suggested organizing workshops and conferences to enhance their involvement in ASPs surveillance [20].

Emerging antimicrobial resistance could be de-escalated by educating and training community pharmacists. The collaboration between healthcare providers is required to improve the implementation of ASPs in a community pharmacy setting in Jordan. Future educational intervention studies are needed to enhance the knowledge and practice of community pharmacists in Jordan.

The main limitation of the study is that interviews were conducted through the Zoom application. Zoom interviews may not give the interviewer clues about how the interview is going, and the interviewer could not read the participants’ body language and preferences, as in face-to-face interviews. Another limitation is the use of pragmatic non-random sample that resulted in the recruitment of a very young population, which could limit study generalizability.

## 5. Conclusions

This study revealed several barriers against the implementation of ASPs in community pharmacies in Jordan. Participants indicated the importance of strict law enforcement at all levels of antimicrobial distribution to improve antimicrobial dispensing and to prevent antimicrobial resistance. Our interviewees also suggested the need for several academic discipline frameworks and activities, proper pharmacist training and good pharmacy practice of antimicrobial guidelines to incorporate ASPs in the community pharmacy settings. This includes additional educational workshops for healthcare providers. Future interventional studies are needed to assess the effects of community pharmacy interventions to improve antibiotic stewardship. Moreover, the implications for pharmacy education are required to be evaluated in a range of pharmacy settings.

## Figures and Tables

**Table 1:** Demographic characteristics of the study sample at baseline (n = 20).

Parameter	Median (IQR)	n (%)
Age (years)	28.5 (8.0)	
Gender		
• Female		14 (70%)
• Male		6 (30%)
Educational level		
• Bpharm		13 (65%)
• MS.C		5 (25%)
• Pharm D		2 (10%)
Job-status		
• Owner		8 (40%)
• Employee		12 (60%)
Community practice experience	4.5 (6.0)	
Site of work		
• Independent community pharmacy		16 (80%)
• Chain community pharmacy		4 (20%)
Place of residence		
• Amman		16 (80%)
• Other		4 (20%)
Number of patients you provide a service to them/day		
• <50		5 (25%)
• ≥50		15 (75%)
Have you ever attended a course/workshop about Antimicrobial Stewardship programs?	27.1 (10.6)	
• Yes		6 (30%)
• No		14 (70%)
Duration of the interview (minute) *		

* Mean(SD), IQR: Interquartile Range.

**Table 2:** List of emerging themes and sub-themes.

Question	Main Theme	Subthemes
Causes of antimicrobial resistance	Lack of appropriate knowledge about antimicrobial agents	----
	Non-restricted prescription of antimicrobials	Lack of law enforcement Malpractice by physician Malpractice by pharmacist Incorrect patients’ beliefs and practice
Perceptions toward ASPs and the role of pharmacists in ASPs	Pharmacists are competent to provide ASPs	----
Barriers against the implementation of ASPs	Organizational obstacles	Lack of law enforcement Hierarchal influence by pharmacy owners
	Resources obstacles	Inadequate resources Lack of time Insufficiently trained pharmacists
	Personal obstacles	Patients’ socioeconomic status Profit concerns by pharmacists
Suggestions to enhance the implementation of ASPs	The need for continues professional development	-----

## Data Availability

The data presented in this study are available in the article.

## Supplementary Materials

The following are available online at https://www.mdpi.com/article/10.3390/antibiotics10040384/s1, Table S1: interview guide.

## References

[1] Shuman E.K., Malani P.N. (2018). Infectious diseases mortality in the United States: Ongoing investment needed for continued progress. JAMA.

[2] Khan M.U., Hassali M.A.A., Ahmad A., Elkalmi R.M., Zaidi S.T.R., Dhingra S. (2016). Perceptions and practices of community pharmacists towards antimicrobial stewardship in the state of Selangor, Malaysia. PLoS ONE.

[3] Cox J.A., Vlieghe E., Mendelson M., Wertheim H., Ndegwa L., Villegas M.V., Gould I., Hara G.L. (2017). Antibiotic stewardship in low-and middle-income countries: The same but different?. Clin. Microbiol. Infect..

[4] Khalil I.A., Troeger C., Blacker B.F., Rao P.C., Brown A., Atherly D.E., Brewer T.G., Engmann C.M., Houpt E.R., Kang G. (2018). Morbidity and mortality due to shigella and enterotoxigenic Escherichia coli diarrhoea: The Global Burden of Disease Study 1990–2016. Lancet Infect. Dis..

[5] Tacconelli E., Carrara E., Savoldi A., Harbarth S., Mendelson M., Monnet D.L., Pulcini C., Kahlmeter G., Kluytmans J., Carmeli Y. (2018). Discovery, research, and development of new antibiotics: The WHO priority list of antibiotic-resistant bacteria and tuberculosis. Lancet Infect. Dis..

[6] Spellberg B., Guidos R., Gilbert D., Bradley J., Boucher H.W., Scheld W.M., Bartlett J.G., Edwards Jr J., America I.D.S.o. (2008). The epidemic of antibiotic-resistant infections: A call to action for the medical community from the Infectious Diseases Society of America. Clin. Infect. Dis..

[7] WHO (2015). Global Action Plan on Antimicrobial Resistance.

[8] Barlam T.F., Cosgrove S.E., Abbo L.M., MacDougall C., Schuetz A.N., Septimus E.J., Srinivasan A., Dellit T.H., Falck-Ytter Y.T., Fishman N.O. (2016). Implementing an antibiotic stewardship program: Guidelines by the Infectious Diseases Society of America and the Society for Healthcare Epidemiology of America. Clin. Infect. Dis..

[9] Dellit T.H., Owens R.C., McGowan J.E., Gerding D.N., Weinstein R.A., Burke J.P., Huskins W.C., Paterson D.L., Fishman N.O., Carpenter C.F. (2007). Infectious Diseases Society of America and the Society for Healthcare Epidemiology of America guidelines for developing an institutional program to enhance antimicrobial stewardship. Clin. Infect. Dis..

[10] Wathne J.S., Kleppe L.K.S., Harthug S., Blix H.S., Nilsen R.M., Charani E., Smith I. (2018). The effect of antibiotic stewardship interventions with stakeholder involvement in hospital settings: A multicentre, cluster randomized controlled intervention study. Antimicrob. Resist. Infect. Control.

[11] Haddadin R.N., Alsous M., Wazaify M., Tahaineh L. (2019). Evaluation of antibiotic dispensing practice in community pharmacies in Jordan: A cross sectional study. PLoS ONE.

[12] Almaaytah A., Mukattash T.L., Hajaj J. (2015). Dispensing of non-prescribed antibiotics in Jordan. Patient Prefer. Adherence.

[13] Raza U.A., Khursheed T., Irfan M., Abbas M., Irfan U.M. (2014). Prescription patterns of general practitioners in Peshawar, Pakistan. Pak. J. Med. Sci..

[14] Rather I.A., Kim B.-C., Bajpai V.K., Park Y.-H. (2017). Self-medication and antibiotic resistance: Crisis, current challenges, and prevention. Saudi J. Biol. Sci..

[15] Suaifan G.A., Shehadeh M., Darwish D.A., Al-Ije H., Yousef A.-M.M., Darwish R.M. (2012). A cross-sectional study on knowledge, attitude and behavior related to antibiotic use and resistance among medical and non-medical university students in Jordan. Afr. J. Pharm. Pharmacol..

[16] Ribeiro J.L., Comarella L. (2015). Bactérias Multirresitentes e Emergência da Resistência Tipo New Delhi Metallo-B-Lactamase-1 (NDM-1). Rev. Uniandrade.

[17] Sarwar M.R., Saqib A., Iftikhar S., Sadiq T. (2018). Knowledge of community pharmacists about antibiotics, and their perceptions and practices regarding antimicrobial stewardship: A cross-sectional study in Punjab, Pakistan. Infect. Drug Resist..

[18] Rehman I.U., Asad M.M., Bukhsh A., Ali Z., Ata H., Dujaili J.A., Blebil A.Q., Khan T.M. (2018). Knowledge and practice of pharmacists toward antimicrobial stewardship in Pakistan. Pharm. World Sci..

[19] Sakeena M., Bennett A.A., McLachlan A.J. (2018). Non-prescription sales of antimicrobial agents at community pharmacies in developing countries: A systematic review. Int. J. Antimicrob. Agents.

[20] Atif M., Asghar S., Mushtaq I., Malik I. (2020). Community pharmacists as antibiotic stewards: A qualitative study exploring the current status of Antibiotic Stewardship Program in Bahawalpur, Pakistan. J. Infect. Public Health.

[21] Abu-Helalah M., Alshraideh H.A., Al-Sarayreh S.A., Al Shawabkeh A.H.K., Nesheiwat A., Younes N., Al-Hader A. (2019). A Cross-Sectional Study to Assess the Prevalence of Adult Thyroid Dysfunction Disorders in Jordan. Thyroid.

[22] Abu Farha R., Abu Hammour K., Mukattash T., Alqudah R., Aljanabi R. (2019). Medication histories documentation at the community pharmacy setting: A study from Jordan. PLoS ONE.

[23] Taber K.S. (2018). The use of Cronbach’s alpha when developing and reporting research instruments in science education. Res. Sci. Educ..

[24] Alefan Q., Halboup A., Fathelrahman A.I., Ibrahim M.I.M., Wertheimer A.I. (2016). Chapter 11—Pharmacy Practice in Jordan. Pharmacy Practice in Developing Countries.

[25] Morisky D.E., Green L.W., Levine D.M. (1986). Concurrent and predictive validity of a self-reported measure of medication adherence. Med. Care.

[26] JFDA (Jordan Food and Drug Administration) (2016). Pharmacovigilance of Medications.

[27] WHO (2018). WHO Provides Support to the Jordanian Ministry of Health in Developing a National Antimicrobial Resistance Surveillance System.

[28] Patton M.Q. (2014). Qualitative Research & Evaluation Methods: Integrating Theory and Practice.

[29] Pope C., Ziebland S., Mays N. (2000). Analysing qualitative data. BMJ.

[30] Wickens H.J., Farrell S., Ashiru-Oredope D.A.I., Jacklin A., Holmes A. (2013). Antimicrobial Stewardship Group of Department of Health Advisory Committee on Antimicrobial Resistance and Health Care Associated Infections (ASG-ARHAI). The increasing role of pharmacists in antimicrobial stewardship in English hospitals. J. Antimicrob. Chemother..

[31] Kalungia A.C., Burger J., Godman B., de Oliveira Costa J., Simuwelu C. (2016). Non-prescription sale and dispensing of antibiotics in community pharmacies in Zambia. Expert Rev. Anti-Infect. Ther..

[32] Gajdács M., Paulik E., Szabó A. (2020). Knowledge, attitude and practice of community pharmacists regarding antibiotic use and infectious diseases: A cross-sectional survey in Hungary (KAPPhA-HU). Antibiotics.

[33] Waseem H., Ali J., Sarwar F., Khan A., Rehman H.S.U., Choudri M., Arif N., Subhan M., Saleem A.R., Jamal A. (2019). Assessment of knowledge and attitude trends towards antimicrobial resistance (AMR) among the community members, pharmacists/pharmacy owners and physicians in district Sialkot, Pakistan. Antimicrob. Resist. Infect. Control Hosp. Epidemiol..

[34] Erku D.A. (2016). Antimicrobial stewardship: A cross-sectional survey assessing the perceptions and practices of community pharmacists in Ethiopia. Interdiscip. Perspect. Infect. Dis..

[35] Appaneal H.J., Luther M.K., Timbrook T.T., LaPlante K.L., Dosa D.M. (2019). Facilitators and Barriers to Antibiotic Stewardship: A Qualitative Study of Pharmacists’ Perspectives. J. Hosp. Pharm..

[36] Bal A.M., Gould I.M. (2011). Antibiotic stewardship: Overcoming implementation barriers. Curr. Opin. Infect. Dis..

[37] Kalungia A., Godman B. (2019). Implications of non-prescription antibiotic sales in China. Lancet Infect. Dis..

[38] Alzoubi K., Al Azzam S., Alhusban A., Mukattash T., Al Zubaidy S., Alomari N., Khader Y. (2013). An audit on the knowledge, beliefs and attitudes about the uses and side-effects of antibiotics among outpatients attending 2 teaching hospitals in Jordan. East. Mediterr. Health J..

