# Supplementary material for: Views of Community Pharmacists on Antimicrobial Resistance and Antimicrobial Stewardship in Jordan: A Qualitative Study
Source: Antibiotics (Basel). 2021 Apr 3;10(4):384. doi: 10.3390/antibiotics10040384 (PMC8067308; doi:10.3390/antibiotics10040384)
Supplement: Supplementary file 1 [file antibiotics-10-00384-s001.pdf]

Table S1. Interview guide

1. As we all know, antibiotics resistance is one of the biggest public health challenges of our time. Many causes lead to microbial resistance, in your opinion, what are the main causes contributed to this problem in Jordan?
2. WHO promotes strategies to avoid the emergence and spread of microbial resistance. One of the most important strategies is the optimal use of antibiotics by applying antimicrobial stewardship program. Antimicrobial stewardship is a coordinated program that promotes the appropriate use of antimicrobials (including antibiotics), improves patient outcomes, reduces microbial resistance, and decreases the spread of infections caused by multidrug-resistant organisms. And WHO said that the pharmacists are part of the team. how do you think the role of the pharmacist come up with the guidelines?
3. What do you think the barriers that would limit the participation of pharmacists on ASPs?
4. Finally, do you have any thoughts/ suggestions to enhance the role of pharmacists in antimicrobial stewardship programs?
